# Case Study: A Bio-Inspired Control Algorithm for a Robotic Foot-Ankle Prosthesis Provides Adaptive Control of Level Walking and Stair Ascent

**DOI:** 10.3389/frobt.2018.00036

**Published:** 2018-04-11

**Authors:** Uzma Tahir, Anthony L. Hessel, Eric R. Lockwood, John T. Tester, Zhixiu Han, Daniel J. Rivera, Kaitlyn L. Covey, Thomas G. Huck, Nicole A. Rice, Kiisa C. Nishikawa

**Affiliations:** ^1^Center for Bioengineering Innovation and Department of Biological Sciences, Northern Arizona University, Flagstaff, AZ, United States; ^2^Department of Mechanical Engineering, Northern Arizona University, Flagstaff, AZ, United States; ^3^BionX Medical Technologies, Inc., Bedford, MA, United States

**Keywords:** biomechanics, level walking, muscle model, powered prosthesis, preflex, stair ascent, trans-tibial amputation

## Abstract

Powered ankle-foot prostheses assist users through plantarflexion during stance and dorsiflexion during swing. Provision of motor power permits faster preferred walking speeds than passive devices, but use of active motor power raises the issue of control. While several commercially available algorithms provide torque control for many intended activities and variations of terrain, control approaches typically exhibit no inherent adaptation. In contrast, muscles adapt instantaneously to changes in load without sensory feedback due to the intrinsic property that their stiffness changes with length and velocity. We previously developed a “winding filament” hypothesis (WFH) for muscle contraction that accounts for intrinsic muscle properties by incorporating the giant titin protein. The goals of this study were to develop a WFH-based control algorithm for a powered prosthesis and to test its robustness during level walking and stair ascent in a case study of two subjects with 4–5 years of experience using a powered prosthesis. In the WFH algorithm, ankle moments produced by virtual muscles are calculated based on muscle length and activation. Net ankle moment determines the current applied to the motor. Using this algorithm implemented in a BiOM T2 prosthesis, we tested subjects during level walking and stair ascent. During level walking at variable speeds, the WFH algorithm produced plantarflexion angles (range = −8 to −19°) and ankle moments (range = 1 to 1.5 Nm/kg) similar to those produced by the BiOM T2 stock controller and to people with no amputation. During stair ascent, the WFH algorithm produced plantarflexion angles (range −15 to −19°) that were similar to persons with no amputation and were ~5 times larger on average at 80 steps/min than those produced by the stock controller. This case study provides proof-of-concept that, by emulating muscle properties, the WFH algorithm provides robust, adaptive control of level walking at variable speed and stair ascent with minimal sensing and no change in parameters.

## Introduction

The development of prostheses is expanding rapidly, resulting in a new generation of robotic devices that behave like the limbs they are designed to replace (Aaron et al., 2006; LeMoyne, 2016). Despite the demonstrable success of the new technologies, significant challenges remain. Compared to intact limbs, state-of-the-art powered prostheses are limited in terms of their speed and adaptability. Foot-ankle prostheses are typically used for either walking (Herr and Grabowski, 2012) or running (McGowan et al., 2012), but not both. Adaptation to changing conditions or variation in terrain remains a significant issue (Farrell and Herr, 2011; Sinitski et al., 2012; Tkach and Hargrove, 2013; Kannape and Herr, 2014). Advances in prosthesis development have been driven largely by technology (e.g., light-weight materials, long-life batteries, programmable electronics, and wireless communication), rather than by advances in understanding of the biological principles underlying human movement.

Powered, ankle-foot prostheses have shown great promise in normalizing gait for people with a unilateral trans-tibial amputation (Aldridge et al., 2012; Sinitski et al., 2012; Agrawal et al., 2013; Gates et al., 2013; Grabowski and D'Andrea, 2013; D'Andrea et al., 2014; Esposito et al., 2014). By assisting users through powered plantarflexion during stance and dorsiflexion during swing, the BiOM T2 prosthesis normalizes metabolic costs, preferred walking speed, and ankle biomechanics (Herr and Grabowski, 2012). However, not all users benefit equally (Gardinier et al., 2017) and many challenges remain, especially for ambulation over varying terrain (Aldridge et al., 2012; Pickle et al., 2016; Russell Esposito et al., 2016).

While provision of motor power permits faster preferred walking speeds than can be produced using only passive devices (Herr and Grabowski, 2012), the use of active motor power raises the issue of control; specifically, when and how much torque assistance to provide under varying terrain conditions (Farrell and Herr, 2011; Tkach and Hargrove, 2013; Kannape and Herr, 2014). State-based control schemes typically depend on pattern recognition algorithms to select among a set of control strategies that may differ among the phases of a particular gait (e.g., stance vs. swing phases of level walking; Au et al., 2007) or among gaits associated with different terrains (e.g., level walking vs. stair ascent; Wilken et al., 2011). It is commonly presumed that, because control approaches typically exhibit no inherent adaptation to varying terrain conditions, some combination of mechanical sensing, manual actuation (e.g., Alimusaj et al., 2009), or other volitional signals (e.g., EMG; Kannape and Herr, 2014) are required to detect the need for a transition from one control strategy to another, and to deliver the appropriate torque for the new conditions (Tkach and Hargrove, 2013).

In contrast, muscles adjust their stiffness instantaneously in response to changes in load (“preflexes”) without requiring input from the nervous system (Nichols and Houk, 1976; Dickinson et al., 2000; Monroy et al., 2007; Nishikawa et al., 2007, 2013). When an applied load stretches a muscle, its stiffness increases to resist overstretch. Likewise during unloading, muscles become more compliant. Muscles behave as non-linear, self-stabilizing springs (e.g., Rack and Westbury, 1974; Richardson et al., 2005), and play an important role in control of movement (Hogan, 1985), particularly in response to unexpected perturbations (Daley et al., 2009; Daley and Biewener, 2011). Although difficult or impossible to test experimentally, it seems likely that muscles contribute generally to motor control (Seiberl et al., 2013, 2015; Hessel et al., 2017).

We recently developed a novel “winding filament” hypothesis for muscle contraction (Nishikawa et al., 2012; Nishikawa, 2016), which provides a biologically plausible mechanism to account for the intrinsic adaptive properties of muscle (Monroy et al., 2007; Nishikawa et al., 2007, 2013). In the winding filament hypothesis, the engagement of the titin spring upon muscle activation provides for a mechanism by which nearly invariant muscle force output can be produced when muscles are activated at varying initial positions (Nishikawa et al., 2013). The winding of titin on the thin filaments upon activation provides for changes in muscle stiffness, not only as a function of muscle recruitment but also in response to applied length changes.

The goals of the present study were to develop a control algorithm based on the winding filament hypothesis (WFH), to implement the algorithm using the powered BiOM T2 foot-ankle prosthesis as a platform, and to test its robustness by comparing performance during level walking at variable speed and stair ascent. In computer science, robustness refers to the property that algorithms perform well not only under the conditions for which they were designed, but also under different conditions that stress the original design assumptions. For this study, we tested the robustness of WFH and BiOM T2 stock controllers by comparing their performance during level walking at variable speed, the task for which their design was optimized, vs. stair ascent, a novel condition with different biomechanical requirements for which the controllers were not explicitly optimized.

## Methods

We first describe the WFH muscle model and the BiOM T2 prosthesis, and next describe our methods for implementing the control algorithm using the BiOM prosthesis as a platform and for subject-specific tuning of each algorithm. We then describe the methods used to compare the performance of the WFH and BiOM stock controllers during level walking and stair ascent, and lastly we describe methods for statistical analysis of data.

### Muscle model based on the winding filament hypothesis

**Figure 1:**
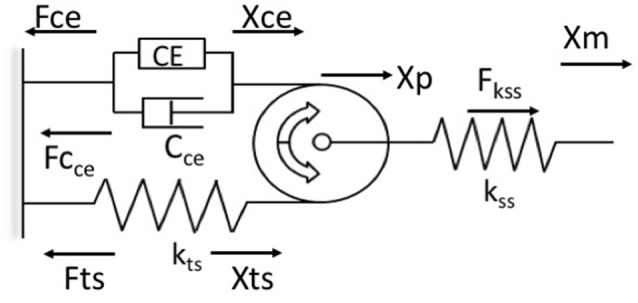
Schematic representation of the muscle model based on the winding filament hypothesis (WFH). The contractile element (CE) is a linear motor with displacement (Xce) and force (Fce). The force velocity relationship of the contractile element is approximated by a viscous damper (C_ce_), with different coefficients for lengthening and shortening. A massless, dimensionless pulley represents the thin filaments in muscle sarcomeres. The pulley translates (X_p_) due to applied forces that stretch or shorten the muscle (X_m_). The titin spring (k_ts_, Fts) is wound around the pulley by activation of the CE and unwinds during deactivation. The titin spring is elongated (X_ts_) by applied forces that translate the pulley toward or away from the contractile element. The series spring (k_ss_, F_ss_) represents muscle tendons, aponeuroses and other series elastic elements. Arrows indicate positive direction. See text for further explanation and equations.

Previous attempts to use bio-inspired neuromuscular control approaches have been limited by the use of Hill models which fail to predict the history-dependence of muscle force (Lee et al., 2013; McGowan et al., 2013). The WFH proposes that muscle cross bridges not only translate but also rotate the thin filaments, which wind titin upon them, storing elastic energy during isometric force development (Nishikawa et al., 2012). In this way, the WFH accounts for history-dependent muscle properties including force enhancement with stretch and force depression with shortening (Nishikawa et al., 2013; Nishikawa, 2016). A muscle model (Figure 1) based on the WFH includes a contractile element (*CE*) that represents myosin cross bridges, a damper (*C*_*ce*_) in parallel with the CE that represents the muscle force-velocity relationship, a pulley that represents actin filaments, and two springs representing titin (*k*_*ts*_) and series elastic elements (*k*_*ss*_).

The contractile element is a linear force generator, similar to the *CE* of Hill muscle models (Zajac, 1989). The *CE* is characterized by an active force-length relationship, *fl(X*_*m*_*)*, where *X*_*m*_ is muscle length, based on overlap between actin and myosin filaments, and a maximum isometric force (*P*_0_) related to muscle cross-sectional area. In the model, the active force-length relationship was measured in mouse soleus muscles and approximated using a second order polynomial (Petak, 2014). Activation of the *CE* results in counter-clockwise rotation of the pulley. This rotation causes the titin spring to wind on the pulley, increasing strain in the free portion of the spring (*X*_*ts*_). Displacement of the titin spring produces a force (*Fts*) that limits further counter-clockwise rotation of the pulley. When the *CE* deactivates, the titin force (*Fts*) rotates the pulley clockwise back to its initial angular position. The parallel damper (*C*_*ce*_) has different coefficients for lengthening (*C*_*ce*_*l*_) and shortening (*C*_*ce*_*s*_), a linear approximation to the lengthening and shortening sides of the force-velocity relationship (Zajac, 1989).

The undamped, linear titin spring (*k*_*ts*_) is connected in series and in parallel to the *CE* via a cable wrapped around the pulley in a no-slip configuration (Figure 1). The force of the titin spring (*Fts*) is modulated by activating or deactivating the contractile element, or by applied forces that displace the pulley toward or away from the *CE*, which decreases or increases the strain in the titin spring, respectively (Figure 1). An undamped, linear spring (*k*_*ss*_) in series with the pulley system (Figure 1) represents the tendon, aponeurosis, and extracellular matrix of the muscle. The series spring (*k*_*ss*_) attaches at the axle of the pulley and is deflected by translation of the pulley but not by its rotation.

The model derivation follows Hooke's law, in which force is the product of the spring deflection (*X*_*i*_) and spring constant (*k*_*i*_). Similarly, the force of the damper is the product of the velocity of the contractile element (ẋ_CE_) and the directional damping constant (*C*_*ce*_*l*_ or *C*_*ce*_*s*_). The balance of forces is calculated by superposition of the two degrees of freedom (translation and rotation) about the pulley (Petak, 2014; Lockwood, 2016). Euler's method is used to calculate the velocity of the damper and the changes in spring lengths at each time step, updated in real time at 500 Hz.

The force of the contractile element (*Fce*) is given by Equation (1):

(1) $$\begin{array}{r} Fce\;=\;act\left(t\right)\;*\;fl\left(X_{m}\right)\;*\;P_{0} \end{array}$$

where activation level, *act(t)*, is an input parameter ranging from 0 to 1, whose value is specified for each stage of the gait cycle and is tuned to user preference (see section Subject-Specific Tuning). The force-length relationship, *fl(X*_*m*_*)* equals 1 at the plateau of muscle optimal length (*L*_0_) and decreases toward 0 at shorter or longer muscle lengths (Zajac, 1989). The peak isometric force (*P*_0_) is also tuned to user preference prior to experimental walking trials.

The rotational force balance around the pulley is given by Equations (2, 3), and the translational force balance is given by Equations (4, 5):

Rotational force balance

(2) $$\begin{array}{r} Fce\;+\;Cce\dot{\text{X}}ce\;=\;ktsXts \end{array}$$

(3) $$\begin{array}{r} \dot{\text{X}}ce\;=\;\frac{ktsXts-Fce}{Cce} \end{array}$$

Translational force balance

(4) $$\begin{array}{r} Fce\;+\;cce\;\dot{\text{X}}ce\;+\;ktsXts=\;kss\left(Xm\;-\;Xp\right) \end{array}$$

(5) $$\begin{array}{r} \dot{\text{X}}ce\;=\frac{kss\left(Xm\;-Xp\right)-\;Fce\;-ktsXts}{Cce} \end{array}$$

where *Fce* is the force of the contractile element, *C*_*ce*_ is the directional damping constant of the contractile element, ẋ_ce_ is the velocity of the contractile element, *k*_*ts*_ is the titin spring constant, *X*_*ts*_ is the titin spring displacement; *k*_*ss*_ is the series elastic spring constant, *X*_*m*_ is the muscle length, and *X*_*p*_ is the pulley displacement. Given input parameters *X*_*m*_ from the BiOM's position sensor and *act(t)* from subject-specific tuning (see section Subject-Specific Tuning), the velocity of the contractile element (ẋ_ce_) is determined by substitution from the sum of rotational (Equations 2, 3) and translational (Equations 4, 5) forces acting on the pulley. The rotational and translational forces are independent and combine using superposition to yield the net force acting on the pulley (Equation 6)

(6) $$\begin{array}{r} Superposition\;\dot{\text{X}}ce\;=\frac{ktsXts\;Fce}{Cce}+\frac{\left[kss\left(Xm\;-\;Xp\right)-\;Fce\;ktsXts\right]}{Cce} \end{array}$$

The derivation assumes equilibrium about the pulley at all times (*t*), and disregards both pulley mass and non-conservative forces (e.g., friction). The muscle model was validated using isokinetic lengthening and shortening data from mouse soleus muscles (Petak, 2014) to demonstrate that it accurately accounts for intrinsic muscle properties, including force enhancement with stretch and force depression with shortening.

### BiOM T2 prosthesis platform

Currently, three types of lower limb prosthetic devices are available commercially for persons with a trans-tibial amputation: passive, quasi-passive, and powered prostheses (LeMoyne, 2016). The BiOM T2 provides powered plantarflexion and dorsiflexion during variable-speed walking (Herr and Grabowski, 2012). It performs negative and positive work by employing a series-elastic actuator comprising a transverse-flux motor and ball-screw transmission in series with a carbon-composite leaf spring (Au et al., 2007; Eilenberg et al., 2010; Markowitz et al., 2011). The motor's rotary motion is converted into linear motion through the ball-screw transmission. The in-series leaf spring improves motor efficiency by storing and returning some of the energy delivered by the motor, storing energy for prosthetic ankle angles < 90° and becoming detached at angles > 90°. A carbon-composite foot at the base of the prosthesis provides additional compliance in the heel and forefoot. The mass of the prosthesis is 2 kg, designed to emulate the mass of a biological foot and partial shank of an 80 kg person (Dempster, 1955). The overall configuration is autonomous; all of the electronic components and the lithium-polymer battery that provides energy to the motor are housed within the prosthesis.

A wireless communication system (Bluetooth) allows for ankle stiffness and power delivery to be adjusted in real time while a person with an amputation walks using the prosthesis. The magnitude and timing of power delivery is calculated within the prosthesis and then adjusted for each wearer to match the performance of a biological ankle during the initial prosthesis fitting. The sensors include motor shaft and ankle joint output encoders, and a 6 degree-of-freedom inertial measurement unit (IMU) comprising three accelerometers and three rate gyroscopes. The BiOM stock controller employs a state-based approach to command ankle torques using a set of algorithms that are implemented in specific stages of the walking gait cycle (early swing, late swing, early stance, mid-stance, late stance). Previous studies demonstrate that the BiOM prosthesis significantly outperforms passive-elastic prostheses, and permits metabolic energy costs, preferred walking velocities and biomechanical patterns over level terrain that are similar to those of people without amputation (Herr and Grabowski, 2012).

### Implementation of the WFH control algorithm using the BiOM prosthesis platform

**Figure 2:**
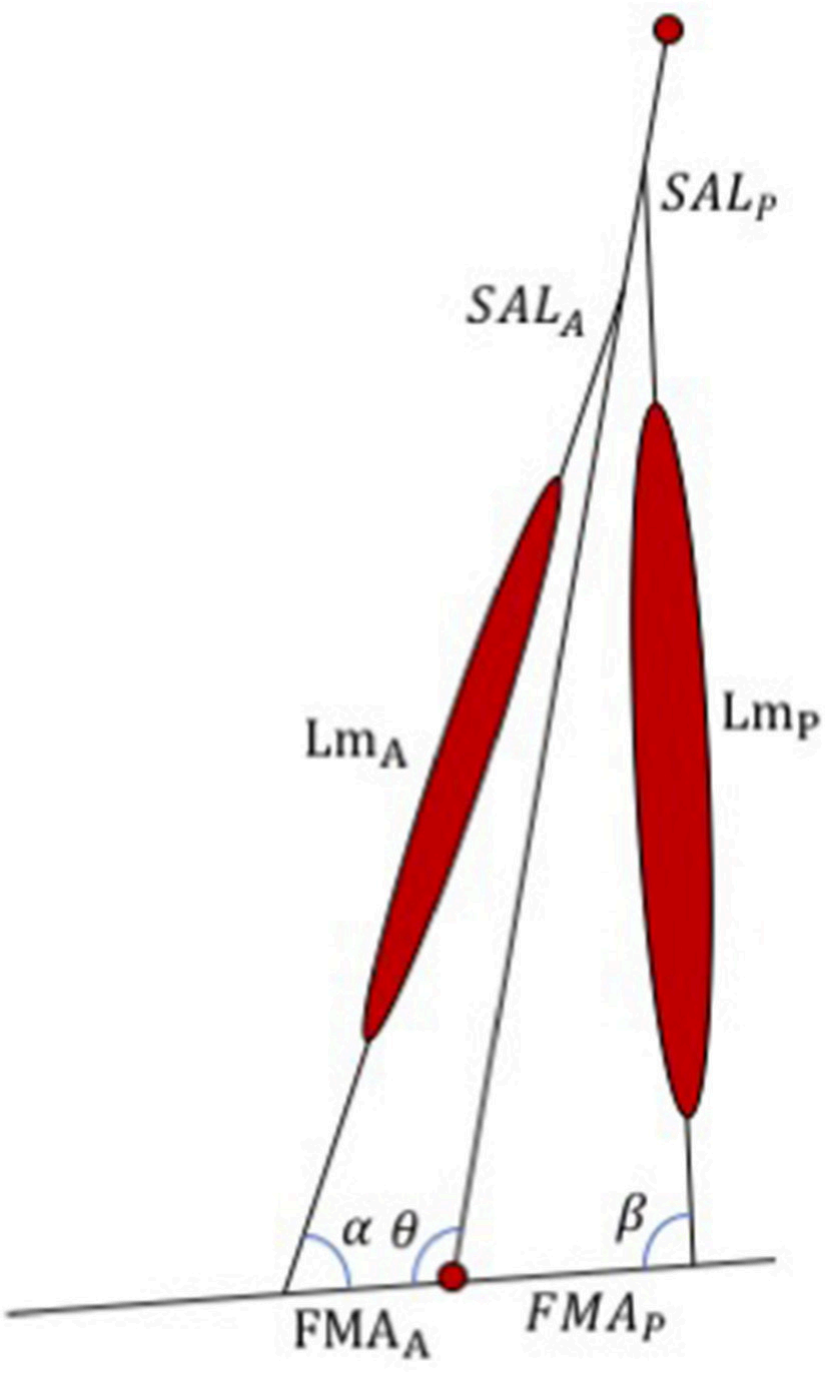
Schematic representation of the WFH algorithm developed for the BiOM prosthesis. The algorithm consists of an anterior and a posterior muscle (represented by WFH muscle models, see Figure 1) with lengths (Lm_A_ and Lm_P_), foot moment arms (FMA_A_ and FMA_P_), shank attachment lengths (SAL_A_ and SAL_P_), and muscle attachment angles (α, β) determined from published values or calculated using geometry (see Table 1). θ = ankle joint angle. See text for further explanation.

The WFH control algorithm was developed in MATLAB and translated to C code to replace the portion of the commercially available BiOM stock controller that determines the motor torque applied to the prosthetic ankle joint. The WFH algorithm (Figure 2) includes a simplified musculo-skeletal model of the shank (tibia/fibula) and foot, using a simple hinge to represent the ankle joint. A pair of virtual muscles provides dorsiflexion and plantarflexion torque, similar to Au et al. (2005). The tibialis anterior and its synergists were approximated as an anterior muscle group (Figure 2) with muscle length (*Lm*_*A*_), shank attachment length (*SAL*_*A*_) and foot moment arm (*FMA*_*A*_). The soleus, gastrocnemius, and plantaris were approximated as a single posterior muscle group (Figure 2) with muscle length (*Lm*_*P*_), shank attachment length (*SAL*_*P*_) and foot moment arm (*FMA*_*P*_). The algorithm thus ignores the biarticular function of the gastrocnemius muscles (Cleather et al., 2015) which attach to the distal femur (Visser et al., 1990). Each virtual muscle group is represented by a WFH muscle model (see Figure 1) scaled using anthropomorphic estimates of length and maximum voluntary force (see Table 1).

**Table 1:** WFH controller parameters established using published values, local optimization, and/or user preference.

**Parameter**	**Definition**	**Value**	**Source**
*fl(X_*m*_)*	Muscle force-length relationship (Equation 1)	0–1	Measured [1]
*P_*o*_A*	Peak isometric force, AM (Equation 1)	1,799 N	[2],[3],[5]
*P_*o*_P*	Peak isometric force, PM (Equation 1)	1,654 N	[2],[3],[5]
*k_*ss*_A*	series spring constant, AM (Figure 1, Equation 6)	1,499 N^*^cm	[4],[5]
*k_*ss*_P*	series spring constant, PM (Figure 1, Equation 6)	1,559 N^*^cm	[4],[5]
*k_*ts*_A*	titin spring constant, AM (Figure 1, Equation 6)	159 N^*^cm	Local optimization
*k_*ts*_P*	titin spring constant, PM (Figure 1, Equation 6)	205 N^*^cm	Local optimization
*C_*ce*_*l*_A*	CE damping constant lengthening, AM (Figure 1)	97 N^*^s/cm	Local optimization
*C_*ce*_*l*_P*	CE damping constant lengthening, PM (Figure 1)	102 N^*^s/cm	Local optimization
*C_*ce*_*s*_A*	CE damping constant shortening, AM (Figure 1)	182 N^*^s/cm	Local optimization
*C_*ce*_*s*_P*	CE damping constant shortening, PM (Figure 1)	57 N^*^s/cm	Local optimization
*Lm_*A*_*	Anterior muscle length (Figure 2)	Variable	Angle sensor input
*Lm_*P*_*	Posterior muscle length (Figure 2)	Variable	Angle sensor input
*SAL_*A*_*	Shank attachment length, AM (Figure 2)	29 cm	[5]
*SAL_*P*_*	Shank attachment length, PM (Figure 2)	33 cm	[5]
*FMA_*A*_*	Foot moment arm, AM (Figure 2)	4 cm	[6],[7]
*FMA_*P*_*	Foot moment arm, PM (Figure 2)	5.5 cm	[5],[7]
α	Attachment angle, AM (Figure 2)	Degrees	Calculated from geometry
β	Attachment angle, PM (Figure 2)	Degrees	Calculated from geometry
θ	Ankle joint angle (Figure 2)	Variable	Angle sensor input
*Act(A2)*	Activation, AM Stage 2 (Equation 1)	0.93, 0.63	User preference
*Act(A3)*	Activation, AM Stage 3 (Equation 1)	0.28, 0.48	User preference
*Act(A4)*	Activation, AM Stage 4 (Equation 1)	0.31, 0.51	User preference
*Act(A5)*	Activation, AM Stage 5 (Equation 1)	0	[8]
*Act(A6)*	Activation, AM Stage 6 (Equation 1)	0	[8]
*Act(P2)*	Activation, PM Stage 2 (Equation 1)	0	[8]
*Act(P3)*	Activation, PM Stage 3 (Equation 1)	0	[8]
*Act(P4)*	Activation, PM Stage 4 (Equation 1)	0	[8]
*Act(P5)*	Activation, PM Stage 5 (Equation 1)	0	[8]
*Act(P6)*	Activation, PM Stage 6 (Equation 1)	0.44, 0.69	User preference

[1] *Petak (2014)*.

[2] *Hoppeler and Flück (2002)*.

[3] *Fukunaga et al. (1992)*.

[4] *Maganaris and Paul (1999)*.

[5] *Arnold et al. (2010)*.

[6] *Maganaris and Paul (1999)*.

[7] *Baxter et al. (2012)*.

[8] *Krishnaswamy et al. (2011)*.

*AM, anterior muscle; PM, posterior muscle. Activation parameters Act(A2-4) and Act(P6) are given for Subject 1 and Subject 2, respectively. All other parameters were the same for both subjects. Modified from Petak (2014)*.

Model parameters were determined using a combination of published values, a local optimization process, simulation-based tuning, and user preference (Table 1). Initial values of peak isometric force (*P*_0_, Table 1) were based on published values of the cross-sectional area of human shank muscles (Fukunaga et al., 1992; Arnold et al., 2010), scaled by 9.5 N/cm^2^ for peak voluntary contraction (Hoppeler and Flück, 2002). Initial parameter values for muscle and tendon lengths, foot moment arms, and attachment angles of the anterior and posterior muscles were determined using published values (Table 1). Maximum isometric muscle force, P_0_, as well as spring and damping constants were optimized using a local optimization function (Matlab FMINCON), and adjusted for best fit to the observed ankle torque data collected during walking trials using the BiOM stock controller.

The ankle joint angle was calculated in real-time using input from the BiOM's ankle angle encoder and shank geometry. The law of cosines allows calculation of virtual muscle lengths from the BiOM's ankle angle encoder. Given virtual muscle length and activation *act(t)* for each muscle, the WFH algorithm calculates the ankle moment of each muscle, and computes net ankle joint moment which, after compensating for mechanical resistance, is sent as a command to the BiOM motor.

The walking gait cycle of humans consists of stance (~60%) and swing (~40%) phases (Vaughan et al., 1990). The BiOM state detection algorithm further distinguishes early swing (state 2), late swing (state 3), early stance (state 4), late stance (state 5), and powered plantarflexion (state 6) on the basis of torque and timing. During human walking, muscle activation patterns differ depending on the stage of walking (Krishnaswamy et al., 2011). The tibialis anterior muscle, which provides ankle dorsiflexion, is activated just prior to toe-off, and remains activated throughout swing and into early stance. The posterior muscles, soleus, and gastrocnemius, are active during the stance phase of walking and silent while the foot is in the air (Lichtwark and Wilson, 2006; Krishnaswamy et al., 2011).

The WFH control algorithm uses the BiOM state machine *only* to provide phase-dependent activation (0–100% of maximal isometric muscle force, P_0_) of the anterior and posterior muscles that approximates biological muscle activation patterns. The anterior muscle group is active (~60–90% P_0_) during early swing, late swing (~30–50% P_0_), and early stance (~30–50% P_0_) and the posterior muscle group is only active (~40–70% P_0_) during powered plantar flexion. The activation levels were adjusted to user preference during tuning sessions preceding experimental trials (see section Subject-Specific Tuning).

A series of simulations was conducted to determine the sensitivity of the model to the parameter values (see Table 1). For a representative level walking trial (Subject 1, 1.65 m/s), the parameter values were varied systematically over a wide range (typically 0.5–250% of the optimal value). The predicted ankle moment for each parameter set was compared to the experimentally observed ankle moment during the representative trial using error analysis and the results reported as R^2^. The sensitivity analysis showed that the WFH algorithm predicts similar ankle torques over a wide range of parameter values. A change of ±25% in parameter values resulted in a <2% change in R^2^ for all variables except *P*_*o*_*P, C*_*ce*_*s*_*P*, and *Act(P6)*, for which R^2^ decreased by 11, 4, and 9% respectively.

### Subject-specific tuning

Two healthy male adults (age 34 and 35 years; height 181 and 184 cm; weight 82 and 109 kg), with traumatic, unilateral trans-tibial amputation 10, and 11 years prior to the study and no neuromuscular disorders or injuries, gave free informed consent to participate in this study. Both subjects had prosthetic ambulation skills for variable cadence, traversing most environmental barriers, and for vocational, therapeutic, or exercise activity that demands prosthetic use beyond simple locomotion (i.e., K3/K4 ambulation).

At the time of the study, both subjects had owned and used a BiOM T2 prosthesis in daily ambulatory activities for 4 and 5 years. Both subjects used their own socket and footplate attached to a BiOM T2 prosthesis specifically modified for this study with a softer hard-stop spring that allowed up to 2° of dorsiflexion and with software enabled to run both stock and WFH controllers.

The BiOM stock and WFH controller parameters were tuned for each subject in three phases during at least two tuning sessions. Subject-specific tuning of the BiOM stock controller used standard operating procedures recommended by the manufacturer and included: (1) accounting for subject mass; (2) adjusting ankle stiffness at heel strike by increasing or decreasing early stance stiffness; and (3) adjusting the power provided at slow and fast walking speeds during powered plantarflexion based on user preference. For the WFH controller, P_0_ and activation levels (0–100%) of the anterior and posterior muscle groups (Table 1) during each phase of walking were determined based on subject preference. Once parameters for the stock and WFH controllers were determined for each subject during level walking, the same parameter values were used in all trials (level walking and stair ascent) for that subject.

### Testing subjects with a trans-tibial amputation

#### Metabolic cost of transport

We measured the metabolic cost of walking with the WFH vs. stock controllers at walking speeds of 0.75, 1.2, and 1.65 m/s. Gross rates of oxygen consumption and carbon dioxide production were measured using a ParvoMedics TrueOne 2400 (Sandy, UT) metabolic cart, while subjects walked on a Woodway Desmo (Waukesha, WI) treadmill. The order of velocities tested was randomized and subjects rested for at least 5 min between trials. Steady-state metabolic power (W) from 4–6 min of each trial was estimated using a standard equation (Brockway, 1987). The metabolic power was divided by each participant's weight and speed to calculate the metabolic cost of transport (J Nm^−1^).

#### Inverse dynamics vs. BiOM torque sensor

Walking kinematics and kinetics were quantified at three speeds (0.75, 1.25, and 1.65 m/s) for each subject using an AccuGait Optimized force-plate (Advance Medical Technology, Inc.), eight Vicon™ cameras, and Nexus 2.3 motion analysis software. An infrared timing system (Brower Timing Systems, Draper, Utah, USA) was used to determine average walking speed. Trials falling ± 0.05 m/s outside of the prescribed speed were discarded. The force-plate was embedded in a 2.9 m walkway. The cameras were operated at 100 Hz, the same rate as the force plate. IR-reflecting markers were placed on the subjects at standard locations (Winter, 1990; Davis et al., 1991) to track limb position. The Nexus 2.3 lower leg plug-in gait dynamics function was used to estimate ankle torque from inverse dynamics. Anthropometric variables were measured for each subject, including body mass (kg), height (mm), left and right leg length (mm), left and right knee width (mm), and left and right ankle width (mm).

For each algorithm, ankle moments estimated using the BiOM torque sensor and inverse kinematics (derived from video and force plate data) were compared at three speeds (0.75, 1.25, and 1.65 m/s). The BiOM's state machine was used to identify strides on the prosthesis side, with each stride starting at one heel-strike event (0% gait cycle) and ending at the next heel-strike event on the prosthetic side. For level walking, 7–12 strides were analyzed for each subject and each controller at each speed (*n* = 55 strides for Subject 1; *n* = 64 strides for Subject 2).

#### Level walking at variable speed

The BiOM's sensors, validated using inverse dynamics (see section Inverse Dynamics vs. BiOM Torque Sensor below) were used to estimate peak ankle moment (Nm/kg), plantarflexion angle during stance (degrees), and peak ankle power (W/kg) in the sagittal plane during level walking at three speeds (0.75, 1.25, and 1.65 m/s) and during stair ascent. Plantarflexion was defined as a negative ankle angle and dorsiflexion as a positive angle, where 0° represents the neutral position in which the foot is ~perpendicular to the shank. Due to noise in the time stamps for the encoded data from the BiOM sensors and variation in the duration of each stride, the raw data were interpolated to obtain 100 data points for each stride based on total stride duration. The interpolated data were used to calculate ankle angular velocity and ankle power.

#### Stair ascent

Subjects were asked to ascend stairs in a step-over-step manner at self-selected speed and at 80 steps per minute. During stair ascent, subjects were asked to land on each step with the ball of the foot, as they naturally do with the intact limb (Kannape and Herr, 2014). To normalize the self-selected speed, subjects were given the prompt “*walk upstairs at a comfortable, safe pace that would allow you to maintain a conversation with someone walking with you*.” Each subject took 2–6 level ground steps with the prosthesis and then transitioned to the first step with their intact limb. After ascending four stairs on the prosthesis side, the subjects continued walking on level ground for another two strides on the prosthesis side and then ascended four more stairs. A total of 11–62 steps was analyzed for each subject, speed and control algorithm (*n* = 179 steps for Subject 1; *n* = 96 steps for Subject 2).

To compare the robustness of stock and WFH algorithms, we defined robustness operationally as maintenance or improvement of prosthesis performance on stairs vs. level walking relative to average values for control subjects with no amputation performing the same tasks as published in Aldridge et al. (2012). For example, the average plantarflexion angle for Subject 1 using the WFH control algorithm was −11.6° during level walking at 0.75 m/s and it increased to −15.9° during stair ascent at 80 steps/min, so the algorithm is robust because the average plantarflexion angle of control subjects with no amputation ascending stairs at 80 msteps/min was −14.7°.

### Statistics

Statistical comparisons were performed using JMP Pro 13 (SAS Institute, Inc.). BiOM stock and WFH controllers were compared separately for each subjects. Peak ground reaction force was compared using two-way ANOVA (α = 0.05) with controller (BiOM stock vs. WFH), walking speed (0.75, 1.25, 1.65 m/s), and controller x speed as the main effects. One-way ANOVA (α = 0.05) was used to compare ankle moment estimates from the BiOM's torque sensor to the estimates based on inverse kinematics within each combination of controller and walking speed. For level walking, peak ankle moment (Nm/kg), plantarflexion angle (degrees), and ankle power (W/kg) were compared using two-way ANOVA (α = 0.05), with controller, walking speed and controller x speed as the main effects. For stair ascent, peak ankle moment (Nm/kg), plantarflexion angle (degrees), and ankle power (W/kg) were compared separately at each speed (self-selected vs. 80 steps/min) using one-way ANOVA (α = 0.05) with controller as the main effect. To compare robustness of stock and WFH algorithms, we used two-way ANOVA with controller (stock vs. WFH), condition (level walking vs. stair ascent) and controller × condition as main effects. The analysis was performed separately for slow (0.75 m/s level walking vs. 80 steps/min stair ascent) and medium speeds (1.25 m/s level walking vs. self-selected speed for stair ascent).

For parametric analyses, assumptions of normality were evaluated using Shapiro-Wilk tests within each combination of controller and speed. Equality of variances was evaluated using Levene tests for normally distributed data and Brown-Forsythe tests for non-normal data. Each data set was tested for normality and all comparisons between controllers for each speed and subject were tested for equality of variances before and after best Box-Cox transformations. For each subject, a total of 9 dependent variables (including vertical ground reaction force; peak ankle moment from BiOM torque encoder vs. inverse dynamics; and sagittal plane peak ankle moment, ankle plantarflexion angle, and ankle power during level walking and stair ascent) were measured for each controller (BiOM stock and WFH) and walking speed (two for stairs and three for level walking), for a total of 48 data sets per subject. Due to persistent violations of normality and homoscedasticity even after transformation (see results), between-controller comparisons were also tested using non-parametric Steel-Dwass tests.

## Results

**Figure 3:**
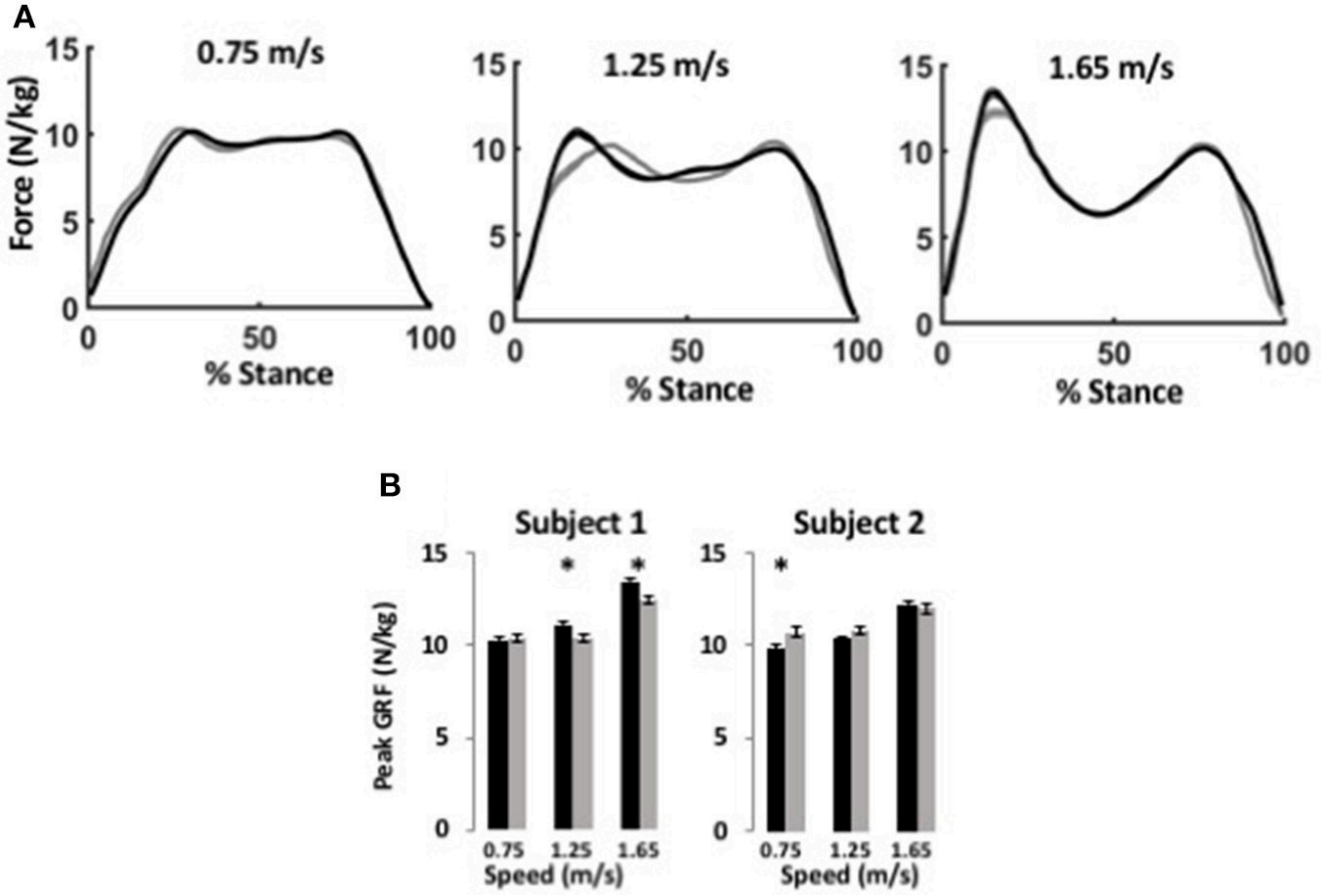
WFH (gray) and BiOM stock (black) controllers produce similar ground reaction forces during level walking at variable speed. **(A)** Average ground reaction force (N/kg) ± SEM vs. % stance for Subject 1 at three speeds. Left, 0.75 m/s; center, 1.25 m/s; right, 1.65 m/s. **(B)** Average peak ground reaction force (N/kg) ± SEM for two subjects. Asterisks (^*^) represent statistical differences (*P* < 0.05) using Steel-Dwass non-parametric comparisons of median values.

**Figure 4:**
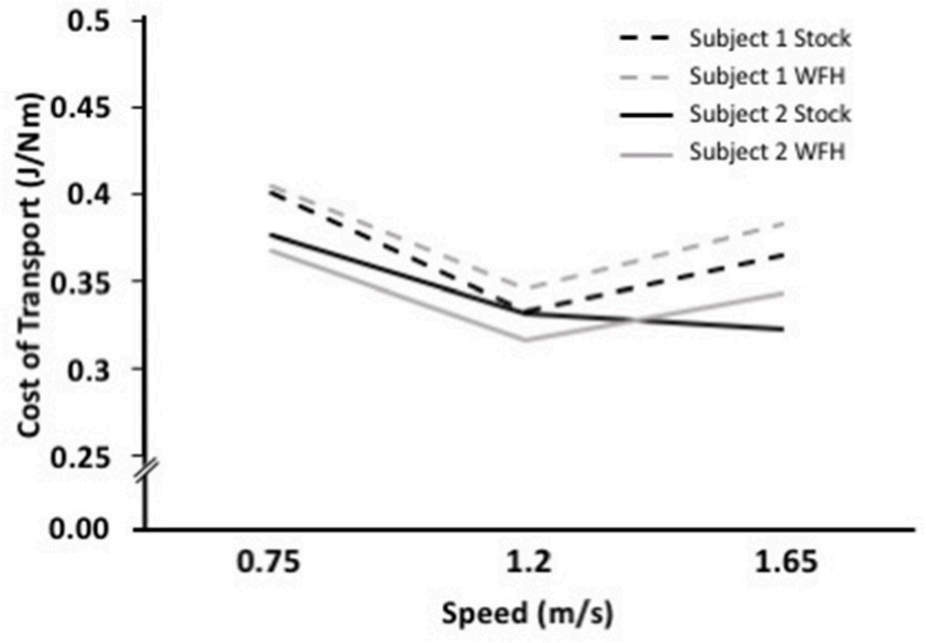
WFH (gray) and BiOM stock (black) controllers produce similar metabolic cost of transport during level walking at variable speed. Cost of transport (J/Nm) for two subjects at three walking speeds (0.75, 1.2, and 1.65 m/s). Broken lines = Subject 1; solid lines = Subject 2.

**Figure 5:**
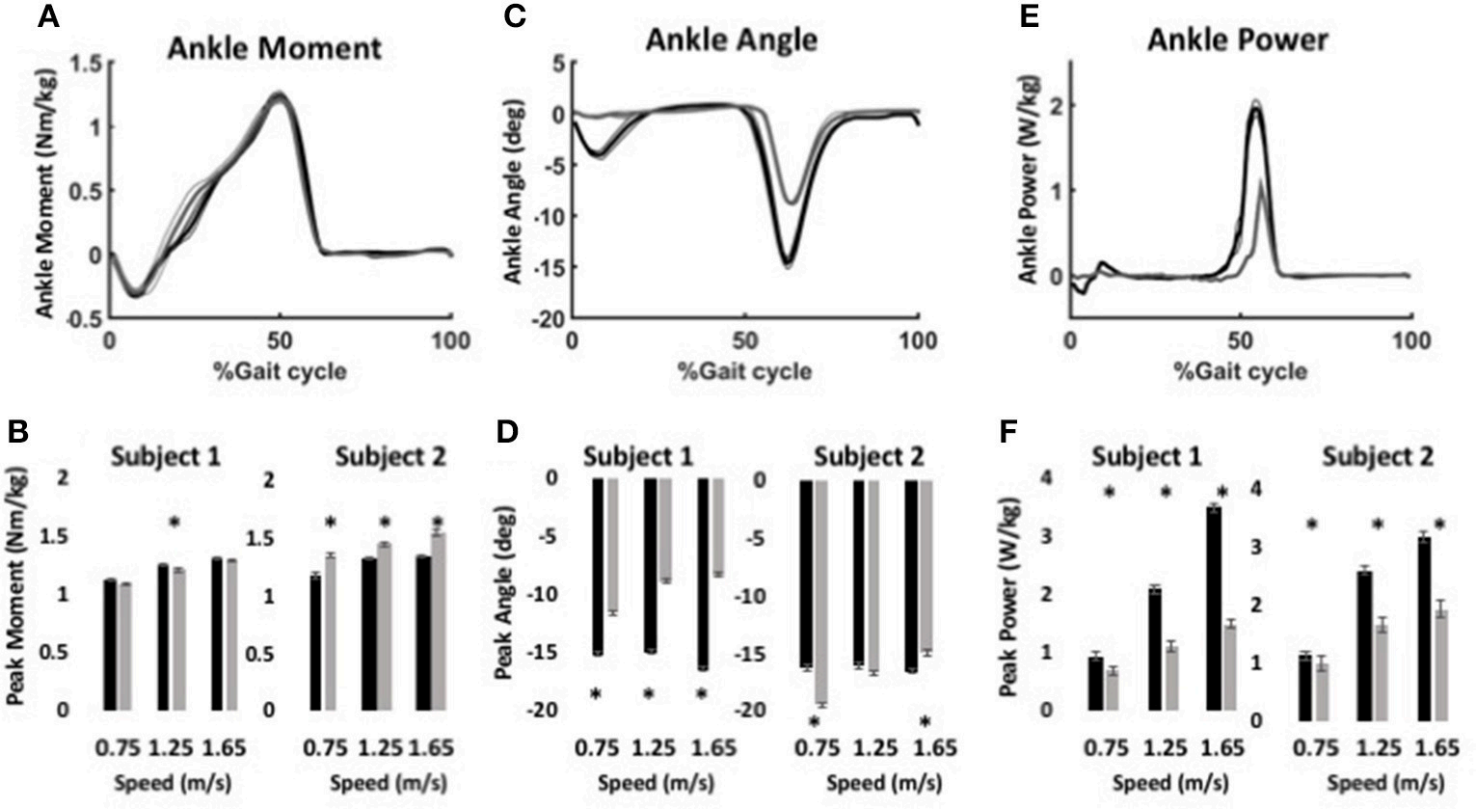
Ankle kinematics and kinetics during level walking at variable speed using WFH (gray) and BiOM stock (black) controllers. **(A)** Average ankle moment (Nm/kg) ± SEM vs. % gait cycle. **(B)** Peak ankle moment (Nm/kg) ± SEM for two subjects at three walking speeds. **(C)** Average plantarflexion angle (degrees) ± SEM vs. % gait cycle. **(D)** Peak plantarflexion angle (degrees) ± SEM for two subjects at three walking speeds. **(E)** Average ankle power (W/kg) ± SEM vs. % gait cycle. **(F)** Peak ankle power (W/kg) ± SEM for two subjects at three walking speeds. Data in **(A,C,E)** are from for Subject 1 walking at 1.25 m/s. Asterisks (^*^) represent statistical differences (*P* < 0.05) using Steel-Dwass non-parametric tests.

**Figure 6:**
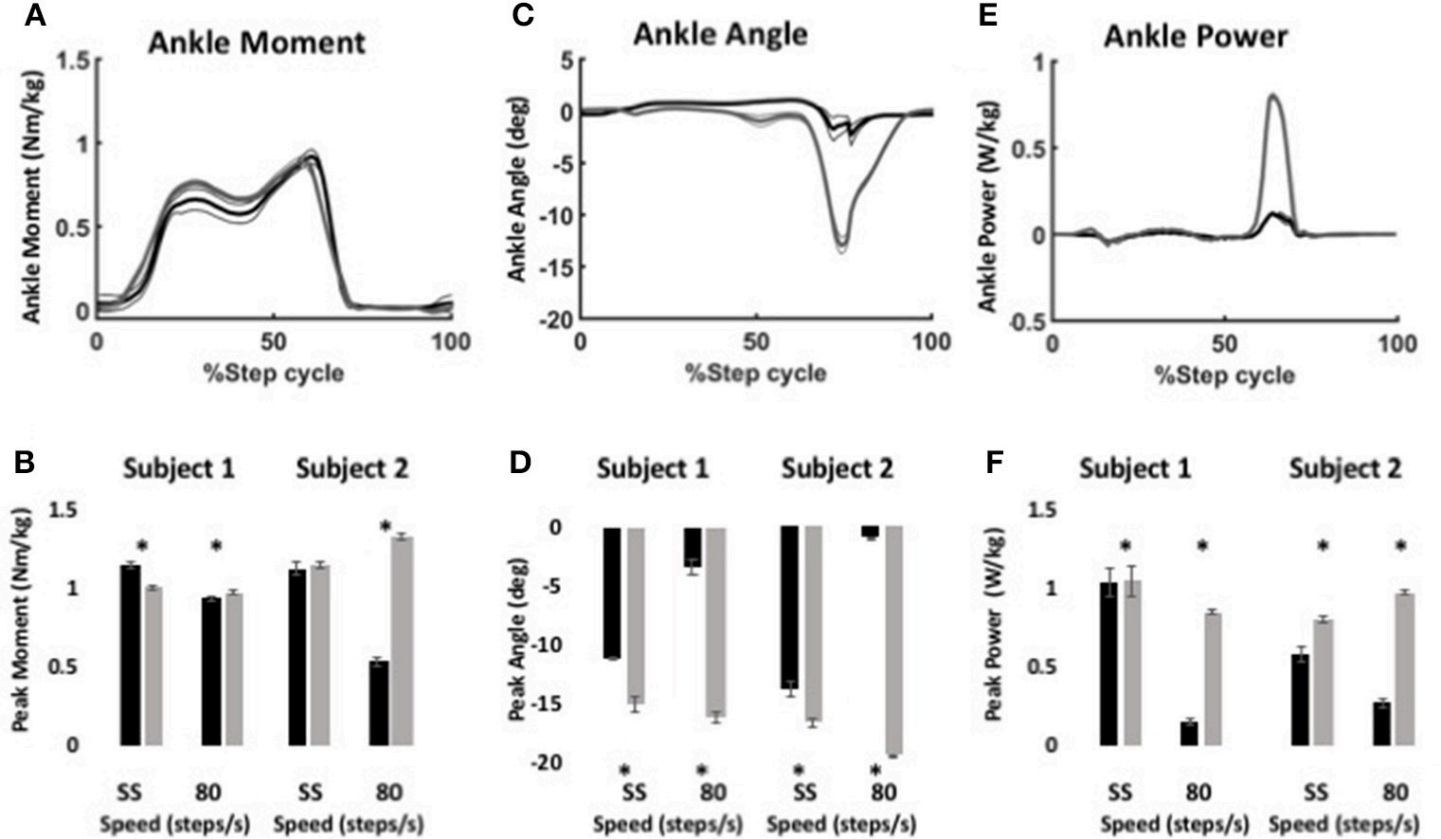
Ankle kinematics and kinetics during stair ascent using WFH (gray) and BiOM stock (black) controllers. **(A)** Average ankle moment (Nm/kg) ± SEM vs. % step cycle. **(B)** Peak ankle moment (Nm/kg) ± SEM for two subjects ascending stairs at self-selected speed and 80 steps/min. **(C)** Average plantarflexion angle (degrees) ± SEM vs. % step cycle. **(D)** Peak plantarflexion angle (degrees) ± SEM for two subjects ascending stairs at self-selected speed and 80 steps/min. **(E)** Average ankle power (W/kg) ± SEM vs. % gait cycle. **(F)** Peak ankle power (W/kg) ± SEM for two subjects ascending stairs at self-selected speed and 80 steps/min. Data in **(A,C,E)** from Subject 2 ascending stairs at 80 steps/min. Asterisks (^*^) represent statistical differences (*P* < 0.05) using Steel-Dwass non-parametric tests.

For Subject 1, 15 of 48 data sets failed the normality test and 10 of 32 comparisons failed the equal variances test after transformation. For Subject 2, 12 of 48 data sets failed the normality test and 11 of 32 comparisons failed the equal variance tests after transformation. ANOVA results are presented in Tables 2–**4**, and results from the more conservative Steel-Dwass tests are indicated by asterisks in Figures 3–**6**. Where ANOVA and non-parametric tests differed, we report the results of the more conservative non-parametric tests.

**Table 2:** Vertical ground reaction force for two subjects.

**Peak GRF (Nm/kg)**	**Subject 1^b^**		**Subject 2^a,b^**	
**Effect**	**F-ratio**	***P*-value**	**F-ratio**	***P*-value**
Speed	161.30	<0.0001	43.94	<0.0001
Controller	11.63	0.0013	3.87	0.0574
Speed x Controller	5.77	0.0057	3.10	0.058

*Results of two-way ANOVA after Box—Cox transformation for stock and WFH controllers during level walking at three speeds (0.75, 1.25, and 1.65 m/s). See Figure 3 for means ± SEM*.

a *Not normally distributed after Box–Cox transformation*;

b *Variances not equal after Box–Cox transformation*.

### Ground reaction force

The BiOM stock and WFH controllers produced similar vertical ground reaction forces (GRF) for both subjects during level walking at all speeds (Figure 3A). For both subjects, peak GRF increased with walking speed (two-way ANOVA, both *P* < 0.0001, Table 2). The subjects differed in effects of the controllers on peak GRF. For Subject 1, peak GRF was 6.7% higher on average for the stock controller at the two faster walking speeds (Figure 3B, Steel-Dwass tests, *P* < 0.0198). For Subject 2, peak GRF was 8.7% higher for the WFH controller at the lowest speed (Figure 3B; Steel-Dwass test, *P* = 0.0065).

### Metabolic cost of transport

The metabolic cost of transport for the two control algorithms differed between subjects. Subject 1 had the lowest cost of transport at all speeds using the stock controller (Figure 4). The WFH-controller performed nearly as well as the stock controller at 0.75 m/s but walking was less efficient with the WFH controller than with the stock controller at the faster speeds. Subject 2 had the lowest cost of transport when using the WFH-controller at the slow and intermediate speeds, but the stock controller had the lowest cost of transport at the fastest speed (Figure 4).

### Inverse dynamics vs. BiOM torque sensor

The accuracy of the BiOM torque sensor was assessed by comparing the peak ankle moment estimated by the sensor to the peak ankle moment estimated from inverse dynamics within each combination of control algorithm (stock vs. WFH) and speed (0.75, 1.25, and 1.65 m/s) for each subject (12 comparisons total). In only one of 12 ANOVA comparisons was there a difference in peak ankle moment between estimates from the BiOM's sensors and inverse dynamics after Box-Cox transformation (Subject 1, 0.75 m/s; *p* = 0.0041). In this case, the BiOM torque sensor underestimated the peak torque by ~8% relative to the estimate from inverse dynamics. Because these results suggest that the BiOM's sensor provide a reliable measure of ankle moment, we used data from the BiOM sensors for subsequent comparisons of algorithm performance.

### Level walking at variable speed

During level walking, maximum forces of the virtual muscle-tendons unit (MTU) were ~800 N for the anterior muscle model and ~1200 N for the posterior muscle model, which are within the range of peak shank muscle forces in observed in human studies (Arnold et al., 2010). Ankle moment profiles were similar for the BiOM stock and WFH control algorithms at all speeds for both subjects (Figure 5A). Peak ankle moment increased significantly with walking speed for both controllers (Figure 5B, two-way ANOVA, *p* < 0.0001, Table 3). Subject 1 achieved a 3.6% higher peak ankle moment during walking using the BiOM stock controller at the intermediate speed (Figure 5B, Steel-Dwass test, *P* = 0.0036), while Subject 2 achieved a 13.2% higher peak ankle moment using the WFH algorithm at all speeds (Figure 5B; Steel-Dwass tests, *P* < 0.017).

**Table 3:** Peak sagittal plane ankle moment, plantarflexion angle, and ankle power for two subjects during level walking at variable speed.

**Peak ankle moment (Nm/kg)**	**Subject 1^a^**		**Subject 2^b^**	
**Effect**	**F-ratio**	***P*-value**	**F-ratio**	***P*-value**
Speed	120.01	<0.0001	109.81	<0.0001
Controller	10.02	0.0027	45.14	<0.0001
Speed x Controller	0.73	0.4857	2.52	0.0896
**Plantarflexion angle (degrees)**	**Subject 1^a^**		**Subject 2^a^**	
**Effect**	**F-ratio**	***P*-value**	**F-ratio**	***P*-value**
Speed	86.63	<0.0001	10.40	0.0021
Controller	2357.59	<0.0001	35.63	<0.0001
Speed x Controller	148.07	<0.0001	47.00	<0.0001
**Ankle power (W/kg)**	**Subject 1^a^^,^^b^**		**Subject 2^a^**	
**Effect**	**F-ratio**	***P*-value**	**F-ratio**	***P*-value**
Speed	366.04	<0.0001	99.82	<0.0001
Controller	356.45	<0.0001	227.89	<0.0001
Speed x Controller	33.82	<0.0001	5.50	0.0066

*Results of two-way ANOVA after Box–Cox transformation for stock and WFH controllers during level walking at three speeds (0.75, 1.25, and 1.65 m/s). See Figure 5 for means ± SEM*.

a *Not normally distributed*;

b *Variances not equal*.

For Subject 1, peak plantarflexion angle achieved during stance (Figures 5C,D) was 38% larger on average for the stock algorithm than for the WFH algorithm at all three speeds (Figure 5D, Table 3; Steel-Dwass tests, *P* < 0.0004). For Subject 2 (Figure 5D), the plantarflexion angle was 20% smaller for the WFH algorithm than the stock algorithm at the slowest speed (Steel-Dwass test, *P* = 0.0001), similar for both controllers at the intermediate speed, and 10% larger for the stock controller at the fastest speed (Steel-Dwass test, *P* = 0.0004). Ankle power (Figures 5E,F) increased with speed for both algorithms (two-way ANOVA, *p* < 0.0001; Table 3) and was significantly larger for the stock algorithm (average 44% for Subject 1 and 29% for Subject 2) for both subjects at all speeds (Figure 5F, Steel-Dwass tests, *P* ≤ 0.0092). Both subjects reported a preference for the stock controller during level walking, which was not surprising based on their extensive previous experience using the BiOM prosthesis with the stock controller.

### Stair ascent

Using the same parameters as for level walking, ankle moment profiles were again similar for the stock and WFH controllers when ascending stairs (Figure 6A). There was no consistent pattern of variation in peak ankle moment (Figure 6B, Table 4) between stock and WFH controllers. For Subject 1, the stock controller had an 12.4% higher peak moment at the self-selected speed (Figure 6B, Steel-Dwass test, *P* < 0.0001), and the WFH controller had a 3.7% higher peak moment at 80 steps/min (Figure 6B, Steel-Dwass tests, *P* ≤ 0.0007). For Subject 2, there was no difference between controllers at the self-selected speed, and the stock controller had a 146% higher peak ankle moment at 80 steps/min (Figure 6B, Steel-Dwass tests, *P* = 0.0001).

**Table 4:** Peak sagittal plane ankle moment (Nm/kg), plantarflexion angle (degrees), and ankle power (W/kg) for two subjects while ascending stairs.

**Variable**	**F-ratio**	***P*-value**	**F-ratio**	***P*-value**
**Self-selected speed**	**Subject 1**		**Subject 2**	
Peak ankle moment (Nm/kg)	34.21^b^	<0.0001	44.64^a^	<0.0001
Plantarflexion angle (degrees)	4.32^a^	<0.0001	11.14^a^	0.0015
Ankle power (W/kg)	13.92^a^^,^^b^	0.0003	34.96^a^^,^^b^	<0.0001
**80 steps/min**	**Subject 1**		**Subject 2**	
Peak ankle moment (Nm/kg)	5.0^a^	0.028	640.27^a^	<0.0001
Plantarflexion angle (degrees)	302.16^a^	<0.0001	68484.5^b^	<0.0001
Ankle power (W/kg)	948.89^a^	<0.0001	584.59	<0.0001

*Results of one-way ANOVA after Box–Cox transformation for stock vs. WFH controllers during stair ascent at self-selected speed and 80 steps/min. See Figure 6 for means ± SEM*.

a *Not normally distributed*;

b *Variances not equal*.

For both subjects at self-selected speed and 80 steps/min, the plantarflexion angle (Figures 6C,D) was significantly greater for the WFH controller than for the stock controller (Figure 6D, Steel-Dwass tests, *P* < 0.0001). The WFH controller increased ankle angle by 35 and 20.2% at self-selected walking speed and by 383 and 1193% at 80 steps/min for Subject 1 and Subject 2, respectively. For both subjects and both speeds, ankle power (Figures 6E,F) was greater for the WFH controller than for the stock controller (Figure 6F, Steel-Dwass tests, *P* < 0.0093), increasing by 0.7 and 39% at self-selected speed and by 255 and 435% at 80 steps/min for Subject 1 and Subject 2, respectively.

**Figure 7:**
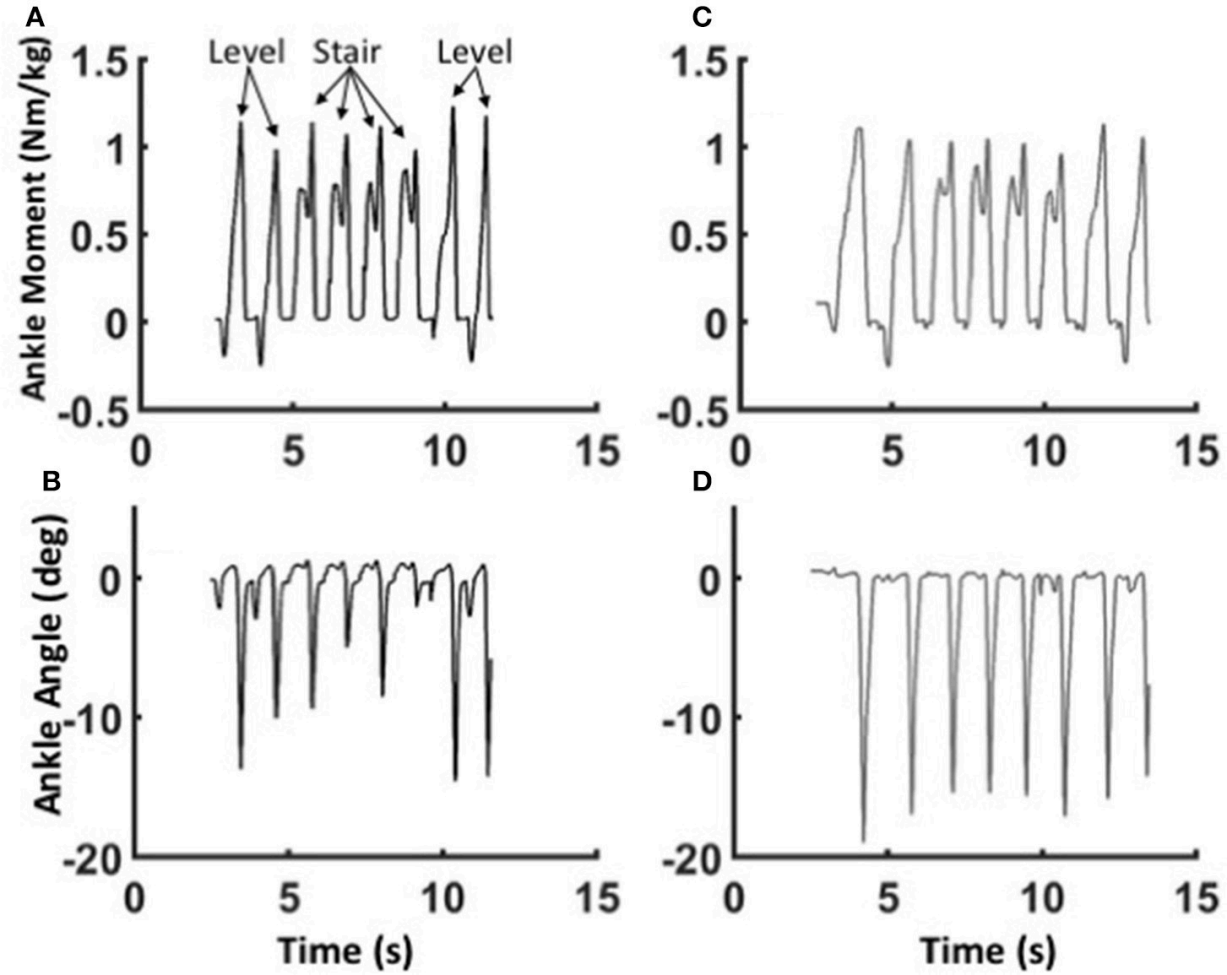
Adaptability of the WFH control algorithm. Ankle moments **(A,C)** and plantarflexion angles **(B,D)** for Subject 1 during the transition from level walking to stair ascent and back to level walking at self-selected speed using the WFH (gray, **A,B**) and stock (black, **C,D**) controllers. Eight consecutive strides are shown in each figure. The subject first takes two strides on level ground, then ascends four stairs on the prosthetic side, and finally takes two level strides at the top of the stairs. For the WFH controller, there is no change in muscle activation or other parameters during transitions from level walking to stair ascent and back to level walking.

The mechanics of level walking and stair ascent are markedly different. In contrast to level walking, stair ascent involves two cycles controlled dorsiflexion and powered plantarflexion; the first cycle pulls the center of mass up from the previous stair, and the second cycle pushes the center of mass up to the next stair (Wilken et al., 2011; Aldridge et al., 2012). Both the BiOM stock and WFH algorithms produced relatively smooth transitions from level steps to stair steps and back to level steps (Figure 7. Pull-up moments in early stance were similar for both algorithms (Figures 7A,C), but push-off moments later in stance were higher relative to pull-up moments for the stock (Figure 7C) compared to WFH controller (Figure 7A). Plantarflexion angles were also smaller and more variable for the stock (Figure 7D) compared to the WFH (Figure 7B) controller, especially during the transitions from level to stairs and vice versa. The WFH controller produced reliable moments and plantarflexion angles during level to stairs transitions and vice versa with minimal sensing (i.e., ankle angle input only) and no change in parameters.

Both subjects expressed a strong preference for the WFH algorithm when ascending stairs. One subject reported that the WFH controller appeared to compensate for the BiOM's weight, so he did not feel that he had to carry the prosthesis up the stairs using his own muscles. Both subjects reported that the WFH algorithm allowed them to ascend stairs in a more natural step-over-step manner than the commercially available stock controller which they had more experience with using.

### Robustness of the WFH algorithm

**Figure 8:**
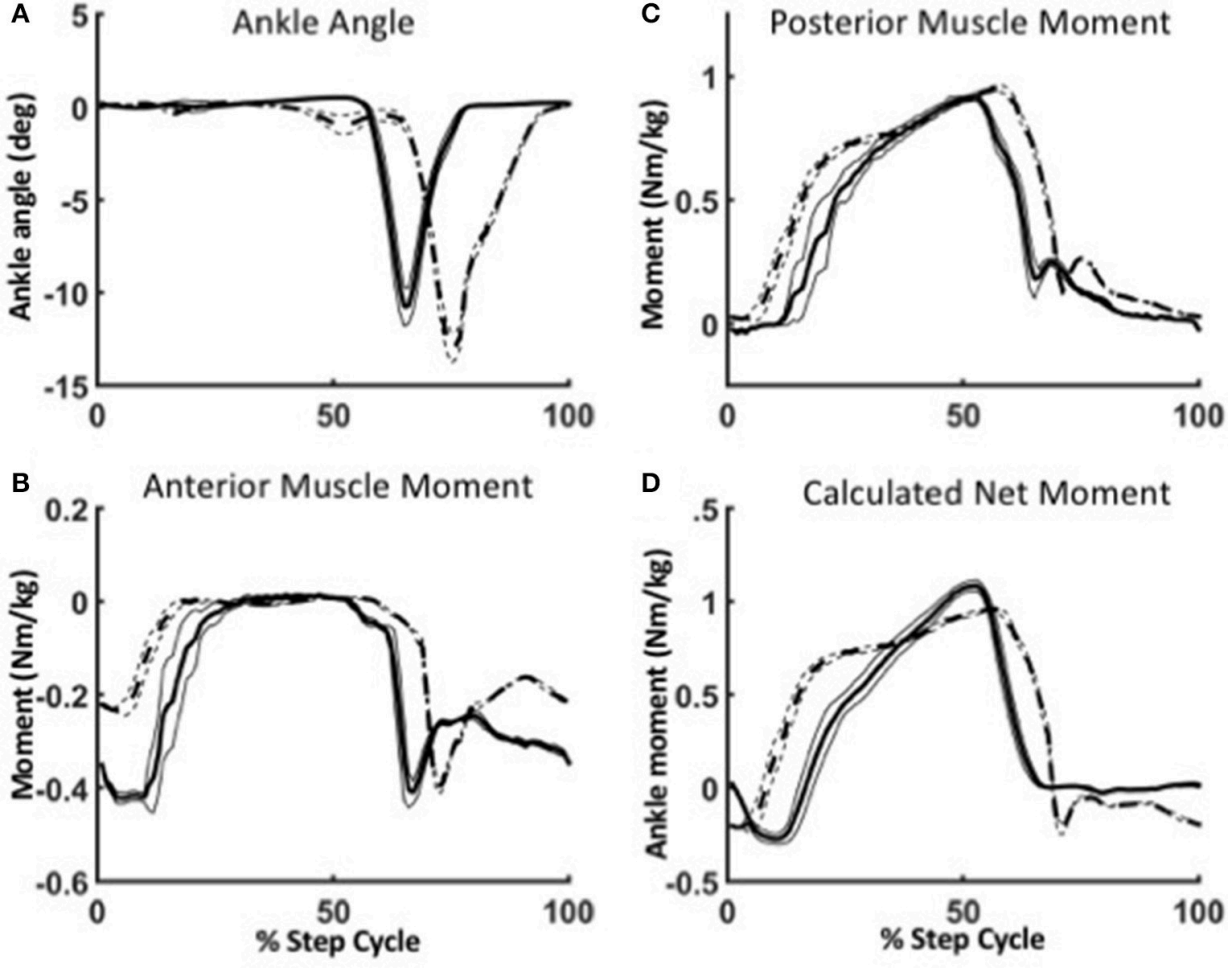
Robustness of the WFH algorithm during level walking (solid lines) and stair ascent (dotted lines). **(A)** Between level walking and stair ascent, the only difference in parameters is the ankle angle. Ankle plantarflexion is larger and occurs later in the gait cycle during stair ascent than during level walking. The change in ankle angle results in different virtual muscle lengths and velocities, which in turn affect the ankle moments produced by the anterior **(B)** and posterior **(C)** muscle models. The net ankle moment **(D)** is the torque command sent to the motor. Lines represent mean ± 1 SEM.

To illustrate the robustness of the WFH algorithm, we evaluated the behavior of the anterior and posterior muscle models during level walking at 1.25 m/s and stair ascent at self-selected speed (Figure 8). The reciprocal lengths of the anterior and posterior muscles are determined strictly by the ankle angle input (Figure 8A) as a function of their moment arms and tendon stiffness. During level walking (solid line), the virtual anterior muscle is stretched during late stance (Figure 8A) and shortens rapidly during early swing, as observed in previous studies during normal walking (Lichtwark and Wilson, 2006). The posterior muscle shows a reciprocal pattern, stretching during swing and shortening during stance to power plantarflexion. During stair ascent, the peak plantarflexion angle was larger and occurred later in the gait cycle than during level walking, as also observed in previous studies (Lichtwark and Wilson, 2006; Spanjaard et al., 2007).

In the WFH algorithm, the net ankle moments produced by the muscle models are the sum of: (1) the contractile element forces (*Fce*) determined by the activation parameters; (2) spring forces determined by a combination of activation and muscle length (*Xm*_*A*_, *Xm*_*P*_); and (3) damping forces determined by the velocity of the contractile element (ẋ_*CE*_). During level walking, the anterior muscle (Figure 8B) produces a dorsiflexion moment during swing, and the posterior muscle (Figure 8C) produces a plantarflexion moment during stance. The net ankle moment (Figure 8D), the sum of anterior and posterior muscle moments, is the command sent to the motor.

The difference in net moment produced by the WFH algorithm during level walking and stair ascent illustrates the algorithm's robustness. The only difference between level walking and stair ascent was the ankle angle input (Figure 8A). There was no change in virtual muscle activation or any other parameters. Yet, the plantarflexion moment produced by the posterior muscle (Figure 8C) rose earlier during stance and was larger during late stance during stair ascent than during level walking. The net torque (Figure 8D) also rose earlier during stance producing a double peak (Figure 8D, dashed line) in net ankle moment typical of normal stair ascent (Sinitski et al., 2012) in contrast to the single peak produced during level walking (Figure 8D, solid line). By emulating muscle intrinsic response to length changes (provided as input via the BiOM ankle angle sensor), the algorithm provides robust control of ankle moment during stair ascent without requiring a change in parameters.

Statistical analysis demonstrated the difference in robustness between WFH and stock controllers in the two subjects who participated in this case study. For this analysis, peak ankle moment, maximum plantarflexion angle, and peak ankle power were compared between the controllers at two speeds (slow and medium) for each subject, for a total of 12 two-way ANOVA tests. A controller was considered to be robust if the change in average value of the dependent variable (e.g., peak ankle moment) either stayed the same during stair ascent or became closer to the average value of that variable observed in control subjects with no amputation performing the same task, published in Aldridge et al. (2012). Of 12 comparisons, both controllers showed robust behavior from level walking to stairs for peak ankle power at medium speed (both subjects) and for peak ankle torque in Subject 2 at medium speed. Neither controller showed robustness for peak ankle torque of Subject 1 at both speeds. For all other comparisons (7/12), the WFH algorithm showed robust performance during stair ascent whereas the stock controller did not. Parameter values of the stock controller moved significantly away from values for control subjects for maximum plantarflexion angle of both subjects at both speeds, for peak ankle torque of Subject 2 at the slower speed, and for peak ankle power of both subjects at the slower speed (two-way ANOVA, *post-hoc t*-tests, all *P* < 0.05).

## Discussion

Although this case study was limited to only two subjects, it represents one of the few studies that includes subjects with extensive experience (4–5 years) using a powered prosthesis for daily activities. Despite the similar history of the two subjects in terms of the time since amputation and experience using the BiOM T2, there were differences between subjects in performance between the stock and WFH controllers during level walking. Subject 1 had a lower cost of transport, larger peak ankle moment, and larger plantarflexion angle during stance when using the stock controller than when using the WFH controller. The reverse was true for Subject 2, for whom the cost of transport was lower, peak ankle moment was larger, and plantarflexion angle during stance was larger at slow and intermediate speeds when using the WFH controller than when using the stock controller. For level walking, the BiOM stock controller produced higher ankle power for both subjects at all speeds. Given their extensive experience using the stock controller, we expected a larger difference in performance between controllers than was observed.

During stair ascent, the WFH controller increased both plantarflexion angle and ankle power for both subjects at the self-selected speed and at 80 steps/min. In a previous study of stair ascent using the BiOM T2 prosthesis, Aldridge et al. (2012) demonstrated that the BiOM T2 prosthesis improved ankle plantarflexion angle by ~10° relative to an elastic storage and return prosthesis at both 80 steps/min (−4.5° vs. +5.8°) and self-selected speed (−4.9° vs. +5.8°). However, even with the BiOM T2 prosthesis, the plantarflexion angle was significantly lower compared to controls with no amputation (−14.7° at 80 steps/min and −15.2° at self-selected speed). In the present study, both subjects achieved ankle plantarflexion angles averaging from −15.1 to −16.7° at self-selected speed and −16.2 to −19.5° at 80 steps/min when using the WFH controller, equivalent to those of control subjects in the previous study (Aldridge et al., 2012). In contrast in the present study, the stock controller produced significantly smaller ankle plantarflexion angles averaging −11.2 to −13.9° at the self-selected speed and −0.9 to −3.4 at 80 steps/min.

The experienced BiOM users in the present study had larger plantarflexion angles during stair ascent at the self-selected speed (−11.2 to −13.9°) than subjects in Aldridge's et al. (2012) study (−4.9° vs. +5.8°), who had acclimated to the BiOM for only ~43days on average. This difference could be due to the greater experience of the subjects in the present study, to the modified hard-stop spring which allows greater dorsiflexion than the commercially available BiOM T2 prosthesis, or both.

Although more ankle power is required to accelerate the center of mass during stair ascent than during level walking (Wilken et al., 2011), it appears that maintaining ankle kinematics is also important for stair ascent. Ankle plantarflexion plays an important role in transitioning off the trailing limb onto the leading limb, and a decrease in ankle plantarflexion requires increased hip extension to raise the center of mass (Aldridge et al., 2012). Although Aldridge et al. (2012) reported no difference in ankle plantarflexor moment or ankle power between the BiOM T2 prosthetic limb and controls with no amputation, the intact limb generated more ankle power than control subjects, and use of the asymmetric “hip strategy,” typically used by people with a unilateral trans-tibial amputation while ascending stairs (Powers et al., 1997; Alimusaj et al., 2009), was not reduced. An important question for future work is whether, by increasing both plantarflexion angle and ankle power during stair ascent compared to the stock controller, the WFH controller can reduce or eliminate use of the “hip strategy,” which increases both gait asymmetry and muscular effort. Increased plantarflexion and ankle power when climbing stairs, as well as smooth transitions between different terrains, may provide a significant improvement in quality of life and cardiovascular fitness for persons with an amputation (Sansam et al., 2009; Sagawa et al., 2011).

The ability of the WFH controller to produce walking at variable speed and stair ascent with minimal real-time sensing (only ankle angle) and no change in virtual muscle activation or other parameters is a significant achievement due to the fundamental differences in gait when ambulating these terrains (Wilken et al., 2011). While the phases of level walking from heel-strike to heel-strike include controlled plantarflexion, controlled dorsiflexion, powered plantarflexion, and swing (Au et al., 2008), the phase transitions during stair ascent include an additional pair of controlled dorsiflexion and powered plantarflexion phases before the swing phase. The first pair of controlled dorsiflexion and powered plantarflexion phases pull the center of mass up from the previous stair, whereas the second pair pushes the center of mass up to the next stair (Wilken et al., 2011; Aldridge et al., 2012). Subjects using the WFH algorithm transitioned smoothly from level walking to stair ascent and vice versa using the same set of equations and parameter values. Ankle kinematics and kinetics were much more variable for both subjects during transitions from level to stairs and back when using the stock controller. The adaptation of ankle torque assistance provided by the WFH controller during gait transitions depends only upon the different ankle angle input, which in turn represents the effects of external forces applied to the virtual muscles at the ankle joint (see Figure 8).

Although many previous studies presume that different operational modes are required for ambulation in different terrains (Tkach and Hargrove, 2013), the present study provides proof of concept that a controller based on muscle intrinsic properties can provide adaptive torque control using the same set of equations and parameters across different gaits and terrains. In principle, this is the same control strategy that animals and humans use during unexpected perturbations when muscles instantaneously adjust their force and stiffness in response to applied length changes long before reflex feedback can modify muscle activation (Daley et al., 2009; Daley and Biewener, 2011). By demonstrating the sufficiency of muscle intrinsic properties for control of level walking and stair ascent, the results suggest the likelihood that these properties may contribute to control of voluntary human movements.

## Limitations

One major limitation of this study is the small sample size of two subjects. Although many previous studies have compared the BiOM T2 prosthesis to passive and quasi-passive prostheses and to control subjects with no amputation, during both level walking (Herr and Grabowski, 2012) and stair ascent (Aldridge et al., 2012; Pickle et al., 2014), a much larger sample is needed to test the repeatability of the results from this small case study. Future studies are also needed to assess whether the WFH algorithm can improve the kinematics and kinetics not only of the affected limb but also the unaffected limb during level walking and stair ascent, as well as regulation of whole body angular momentum.

There are also some limitations associated with the design of the WFH control algorithm that could be addressed in future studies. The first is use of square-waves to simulate activation of the virtual muscles during the different phases of the gait cycle. The sensitivity analysis showed that square-wave activation of the virtual muscles at specific phases during the gait cycle results in fluctuation of the damping forces when muscle activation changes abruptly. These fluctuations in simulated muscle force reduce dorsiflexion during swing at the stance-swing transition, and also increase plantarflexion moment in early stance, which may decrease efficiency and performance of the WFH controller during level walking. Future work should include development of activation strategies that reduce discontinuities in muscle activation across the gait cycle. In the long term, a control strategy for virtual muscle activation that eliminates the requirement for state-based control and provides volitional control, such as EMG from the residual limb, would also likely improve performance and energy efficiency.

## Conclusion

The results of this case study of two experienced BiOM users provide proof-of-concept that a WFH control algorithm based on muscle intrinsic properties can produce ankle kinematics and kinetics during level walking at variable speed and stair ascent that are similar to those produced by the BiOM's stock control algorithm and by people with no amputation. The robust WFH controller transitions from level walking to stairs and vice versa with no change in muscle activation or other parameters, and without requiring information about the user's intended activity. Future work should address optimization of algorithm performance and assessment of its impact on the kinematics and kinetics of the ankle, knee, and hip on affected and unaffected sides, as well as whole-body biomechanics.

## Ethics statement

This study was carried out in accordance with the recommendations of Ethical Principles and Guidelines for the Protection of Human Subjects in Research and Northern Arizona University's Institutional Review Board for the Protection of Human Subjects. All subjects gave written informed consent in accordance with the Declaration of Helsinki. The protocol was approved by the Institutional Review Board for the Protection of Human Subjects at Northern Arizona University.

Animal studies were carried out in accordance with the recommendations of the United States Department of Agriculture (USDA) Animal Welfare Act and Regulations (AWA), the Guide for the Care and Use of Laboratory Animals, Public Health Services Policy on Humane Care and Use of Laboratory Animals, Occupational Safety and Health Administration (OSHA), and Environmental Protection Agency (EPA) regulations, Northern Arizona University's Institutional Animal Care and Use Committee policies and procedures. The research was approved by Northern Arizona University's Institutional Animal Care and Use Committee.

## Author contributions

The experiments were designed by KN, ZH, and JT. UT, AH, and ZH: recruited subjects. UT, EL, AH, KC, and DR: improved the experimental design, performed the experiments, and collected data. UT, EL, KC, NR, and DR: analyzed data using custom code. UT, NR, and TH: prepared the figures. UT, JT, and KN: prepared the first draft of the manuscript. All authors reviewed and edited the manuscript.

### Conflict of interest statement

The authors declare that the research was conducted in the absence of any commercial or financial relationships that could be construed as a potential conflict of interest.
